# Hippocampal damage and memory impairment in congenital cyanotic heart disease

**DOI:** 10.1002/hipo.22700

**Published:** 2017-01-31

**Authors:** Mónica Muñoz‐López, Aparna Hoskote, Martin J. Chadwick, Anna M. Dzieciol, David G. Gadian, Kling Chong, Tina Banks, Michelle de Haan, Torsten Baldeweg, Mortimer Mishkin, Faraneh Vargha‐Khadem

**Affiliations:** ^1^ Cognitive Neuroscience & Neuropsychiatry SectionUCL Great Ormond Street Institute of Child HealthLondonUnited Kingdom; ^2^ Cardiac Intensive Care Great Ormond Street Hospital NHS Foundation TrustLondonUnited Kingdom; ^3^ Developmental Imaging and Biophysics SectionUCL Institute of Child HealthLondonUnited Kingdom; ^4^ Department of NeuroradiologyGreat Ormond Street Hospital for Children NHS Foundation TrustLondonUnited Kingdom; ^5^ Laboratory of NeuropsychologyNational Institute of Mental HealthBethesdaMaryland; ^6^ Department of NeuropsychologyGreat Ormond Street Hospital for Children NHS Foundation TrustLondonUnited Kingdom; ^7^ Present address: UCL Experimental PsychologyLondonUnited Kingdom

**Keywords:** congenital heart disease, transposition of the great arteries, perinatal hypoxia‐ischaemia, memory, hippocampus

## Abstract

Neonatal hypoxia can lead to hippocampal atrophy, which can lead, in turn, to memory impairment. To test the generalizability of this causal sequence, we examined a cohort of 41 children aged 8‐16, who, having received the arterial switch operation to correct for transposition of the great arteries, had sustained significant neonatal cyanosis but were otherwise neurodevelopmentally normal. As predicted, the cohort had significant bilateral reduction of hippocampal volumes relative to the volumes of 64 normal controls. They also had significant, yet selective, impairment of episodic memory as measured by standard tests of memory, despite relatively normal levels of intelligence, academic attainment, and verbal fluency. Across the cohort, degree of memory impairment was correlated with degree of hippocampal atrophy suggesting that even as early as neonatal life no other structure can fully compensate for hippocampal injury and its special role in serving episodic long term memory. © 2017 Wiley Periodicals, Inc.

**Abbreviations**
AHRF: acute hypoxaemic respiratory failure
ASO: arterial switch operation
CSF: cerebrospinal fluid
IQ: intelligence quotients
IVS: intact ventricular septum
TGA: transposition of the great arteries
VSD: ventricular septal defect

## INTRODUCTION

The vulnerability of the hippocampus and, consequently, of memory, to hypoxia/ischaemia has been reported not only in adults (Volpe and Hirst, 1983; Volpe et al., 1986; Zola‐Morgan et al., 1986; Press et al., 1989; Rempel‐Clower et al., 1996; Caine and Watson, 2000; Yonelinas et al., 2004; Allen et al, 2006; Di Paola et al., 2008), but also in children (Vargha‐Khadem et al., 1997; Gadian et al., 2000; Isaacs et al., 2003; De Haan et al., 2006; Golan and Huleihel, 2006). In a recent study (Cooper et al, 2015), we found that a cohort of children with acute hypoxaemic respiratory failure had impaired episodic memory that was correlated with the degree to which their hippocampi had atrophied. Because the children had been selected on the basis, not of memory impairment nor of hippocampal damage, but of the presumed precipitating event, the findings provided especially strong evidence of a causal sequence: hypoxia followed by hippocampal damage followed by memory deficiency. To determine whether this causal sequence was generalisable to a hypoxic‐ischaemic event with a different aetiology, we undertook a study of another cohort of children, this one having been treated at birth for transposition of the great arteries (TGA).

Children with TGA suffer significant cyanosis at birth and thus are potentially at risk of hypoxic/ischaemic damage. Corrective surgery, usually performed within the first week of life using the arterial switch operation (ASO) described by Jatene in 1975 (Jatene et al., 1975), restores ventriculo‐arterial concordance and offers optimal long‐term survival. However, there is an inevitable period of hypoxia/ischaemia prior to the switch operation and another potential period during the operation. With ASO having markedly improved the possibility of survival in cases with TGA, it has become important to assess the risk of brain damage and neuropsychological impairment (von Rhein et al., 2014), with their implications for the survivors' quality of life (Tyagi et al., 2014).

A substantial proportion of survivors of congenital heart disease, including TGA, have been shown on long‐term follow‐up to have below average intelligence quotients (IQ) and reduced academic achievements (Rogers et al., 1995; Kern et al., 1998; Majnemer and Limperopoulos, 1999; Bellinger et al., 2001, 2003; Karl et al., 2004; Freed et al., 2006; Majnemer et al., 2006; Wray, 2006; Creighton et al., 2007; Owen et al., 2013). For example, children with TGA included in the Boston longitudinal study and assessed neuropsychologically at the ages of one, four, eight, and sixteen years (Bellinger et al., 2003, 1999, 2011, 1995), showed a high incidence of learning disabilities, speech and language disorders, and behavioral problems requiring special services (see further reports in Gonzalez and Miller, 2006; Neufeld et al., 2008; Skinner et al., 2008). The Boston study also showed that intelligence, reading, mathematics, and motor function at age eight were adversely affected if the duration of circulatory arrest during surgical repair exceeded about 40 minutes (Wypij et al., 2003). However, cognitive abilities in the majority of neurologically intact patients with TGA were observed to be normal despite the known vulnerability of some brain structures, particularly the hippocampus, to hypoxia/ischaemia. Therefore, although previous studies had suggested that cognitive abilities were preserved in a large proportion of patients with this cardiac condition (Hövels‐Gürich et al., 2001) given the clear risk of systemic hypoxia/ischaemia in these cases, we set out to determine whether they had sustained hippocampal pathology and associated memory impairment.

## METHODS

### Participants

**Figure 1:**
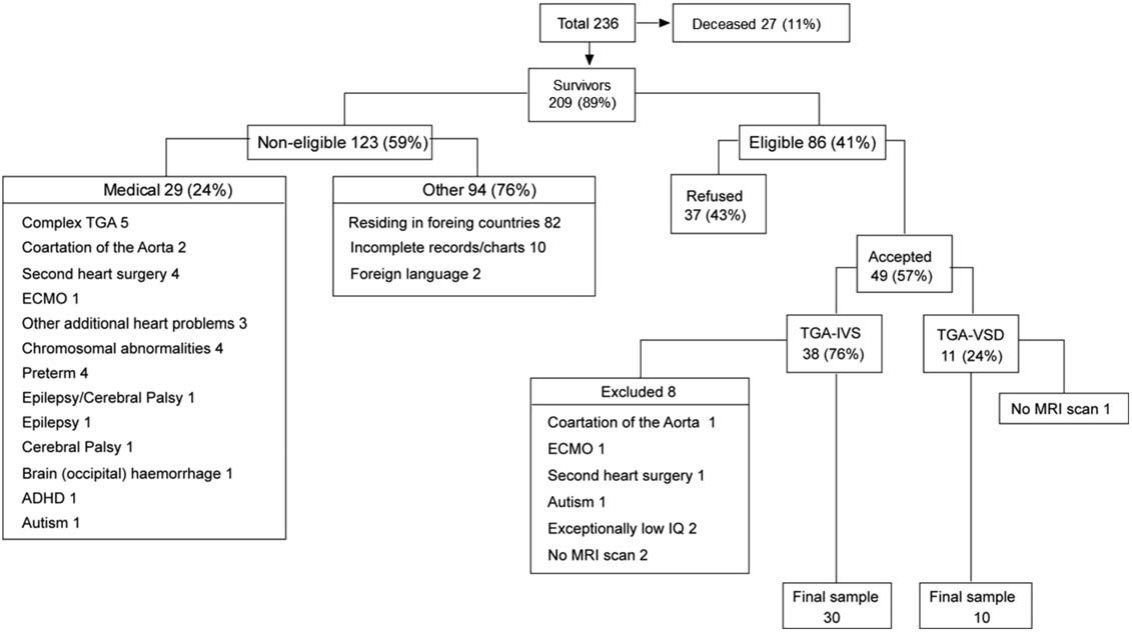
Flow chart illustrating the recruitment of participants.

Forty patients (*M*_age_ = 11.4 years, SD = 2.7; 28 male) participated in the study. As neonates with TGA, they had each undergone the corrective arterial switch operation (ASO) between 1991 and 2000. Participants were selected from a larger group of 236 neonates with TGA (Fig. 1), operated within that same period according to the following inclusion/exclusion criteria: (1) 8–16 years of age at the start of the study; (2) no overt neurological impairment or diagnosis of motor or epileptic disorder, nor learning, or cognitive difficulties; (3) free of genetic syndromes; and (4) native English speakers residing in the UK. Of the final sample of 40 patients, 10 had ventricular septal defect (VSD) and 30 had intact ventricular septum (IVS). Information about the integrity of the ventricular septum was not available in the case of 1 patient.

Sixty‐four healthy children (NC group; *M*_age_ = 10.9 years, SD = 2.1; 28 male; recruited through local schools provided control data for the structural MRI study as well as norms for behavioural tests lacking standard means. Assessment of the patients′ socioeconomic status was based on parental occupation (Ganzeboom et al, 1992) and determination of their behavioural and social problems were based on parental ratings using the Child Behavior Checklist (Achenbach, 1991).

The study was approved by the Research Ethics Committee of University College London Hospital (Reference 05/Q0502/88). Both the children and their parents gave written informed consent and/or assent as appropriate.

### MRI Protocol for Assessment of Brain Integrity

Whole‐brain MRI scans were obtained using a 1.5‐T Siemens Avanto scanner, with a T1‐weighted 3D FLASH sequence: repetition time 11 ms, echo time 4.94 ms, flip angle 15°; matrix size, 224 × 256; field of view, 250 mm; partition thickness, 1 mm; 176 sagittal partitions in the third dimension; acquisition time, 5.34 min. Left, right, and bilateral hippocampal volumes, and total brain grey matter (GM), white matter (WM), and cerebrospinal fluid (CSF) volumes were obtained from the unified segmentation procedure implemented in SPM8 (http://www.fil.ion.ucl.ac.uk/spm/software/spm8/). All volume measurements were corrected for intracranial volume. The scans were screened for any visually detectible signs of extrahippocampal lesions. Based on the judgement of signal abnormality and volume loss, a rating of 0 indicated absence, and 1 signalled the presence of a lesion. Severity was rated on a scale from 0‐2, where 0 was mild; 1 moderate; and 2 severe.

Hippocampal volumes (HV) were obtained as described before (Cooper et al., 2015). Briefly, the 3D datasets were reformatted to 1‐mm‐thick contiguous slices in a tilted coronal plane perpendicular to the caudo‐rostral length of the hippocampus using MEDx 3.43 (Medical Numerics, Inc., Maryland, USA), and cross‐sectional areas were measured (blind to the behavioral data) along the length of the hippocampus and included the dentate gyrus, the hippocampal CA1‐CA3 fields, subiculum, parasubiculum, and presubiculum.

### Neuropsychological Protocol for Cognitive Assessment

The participants were assessed on the Children's Memory Scale (CMS, Cohen, 1997) which provides measures of immediate and delayed verbal and visual memory, yielding a general memory index denoted as memory quotient (MQ), as well as measures of learning, delayed recognition, and attention. Event and spatial memory were assessed with the Rivermead Behavioural Memory Test (RBMT‐II, Wilson et al., 1985) or, for participants aged 8:0–10:11, with the children's version of that test (RBMT‐C, Wilson et al., 1991). The orientation question (i.e. date), which is absent in the children's version, was eliminated from the adult version, so that both tests had the same maximum final profile score of 22. In addition, parents rated their children on a child‐adapted survey of everyday memory and spatial navigation (the Sunderland Questionnaire, Sunderland et al., 1983) and on the Child Behaviour Check‐List (CBCL, Achenbach 1991).

The participants were also assessed on tests of intelligence, academic attainments, and verbal fluency using the Wechsler Intelligence Scale for Children – Version IV (WISC‐IV, Wechsler, 2003), the Wechsler Individual Achievement Test ‐II (WIAT‐II, Wechsler, 2001) and the Letter and Category Fluency subtests of the Delis‐Kaplan Executive Function System (Delis et al., 2001), respectively.

### Statistical Analyses

Statistical analyses, conducted using SPSS version 21, were preceded by the Kolmogorov‐Smirnov test of normality. Control‐patient comparisons of brain‐region volumes and cognitive measures were conducted with independent sample t‐tests, and Pearson correlations were run between brain measures and cognitive scores, with one exception. Because the RBMT data did not distribute normally, the two groups' scores on this test were compared instead with, the Mann‐Whitney U test, and the nonparametric, Spearman correlation was run between the brain volumes and cognitive scores.

## RESULTS

Indices of parental socioeconomic status, as well as parental ratings of the patients' behavioural and social profiles were within normal range (Table 1).

**Table 1:** Indices of Parental Socioeconomic Status and Parental Ratings of the Patients' Behavioral and Social Profiles

	N	Mean	SD	Range
Socioeconomic status^a^				
Father's occupation	36	48.78	17.0	23–77
Mother's occupation	33	52.52	14.1	16–77
Child behaviour checklist (CBC)^b^				
Anxious‐depressed	38	55.45	7.4	50–80
Withdrawn‐depressed	38	54.39	5.4	50–70
Somatic complaints	38	56.16	7.5	50–80
Social problems	38	55.53	5.6	50–70
Thought problems	38	55.26	6.2	50–72
Attention problems	38	56.84	6.7	50–75
Delinquent behaviour (rule‐breaking)	38	53.82	4.6	50–64
Aggressive behaviour	38	53.26	4.3	50–68

a Parental occupation classified according to the International Standard Classification of Occupation (ISCO‐88) transformed into an International Socioeconomic Index of Occupational Status (Ganzeboom et al. 1992) to provide socioeconomic values for the parental occupations with a range of 0–90 (mothers' occupation. *P =* 0.109; fathers' occupation *P =* 0.073).

b The Child Behavior Checklist (CBC; Achenbach 1991), designed to assess behavioral and social problems of children and adolescents as judged by parental ratings, uses *t*‐scores with a mean of 50 (SD 10) where < 60 = normal range of functioning; 60–70 = borderline range; and > 70 = clinical range.

Abbreviation: SD, standard deviation.

### Brain Integrity and Neuropsychological Function in the TGA Group

#### MRI indices of brain integrity

**Figure 2:**
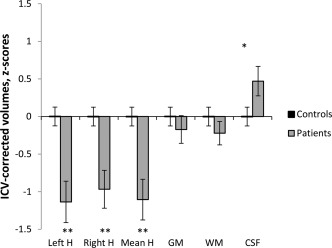
ICV‐corrected hippocampal and global brain volumes in 40 TGA patients against a mean of 64 controls (z‐scores). *denotes p‐values significant at 0.05 level (independent samples t‐test, df = 103). **denotes p‐values significant at 0.001 level (independent samples t‐test, df adjusted).

Global brain measures, such as total grey matter and white matter volumes, in the TGA group were at control levels (Fig. 2). In contrast, there was a, selective, bilateral reduction in HVs in the TGA group (TGA mean, 2966.31 mm^3^; NC, 3248.64 mm^3^, *P* < 0.001; Fig. 2). The patient group also showed a small but significant increase in cerebrospinal fluid (CSF) (TGA mean, 276.5 cc; NC, 271.74 cc; *P* = 0.036), presumably a reflection of the hippocampal atrophy.

Qualitative examination of the MRI showed neuroradiological abnormalities in 14/40 (35%) patients, with the ratings in each case reported as mild (i.e. a score of 0). The findings included deep white matter scars or subtle periventricular leucomalacia (8), right polymicrogyria (1), reduced volume of parieto‐occipital white matter (1), small splenium (1), bilateral plagiocephaly (1), small bilateral cortical and subcortical scars (1), large right lateral ventricle (1). Basal ganglia were reported as normal in all the patients. No significant abnormalities were detected in the control group.

#### Neuropsychological function

Whereas the TGA group scored at the same level as the normal population (standard mean [SM] = 100, SD = 15) on the measures of intelligence, academic attainments, and verbal fluency (with one exception: processing speed), they were deficient on tests sensitive to hippocampal dysfunction (i.e. MQ and delayed verbal and visual memory), scoring significantly below the standard mean on these measures (Table 2). In addition, their profile scores on the test of episodic memory (RBMT) fell below those of the NC group (Median = 18 vs. 21 *P <* 0.001), a finding indicative of memory difficulty for the activities of daily life, including spatial memory.

**Table 2:** Cognitive Profile of Children Who Had the Arterial Switch Operation for TGA (age at test: 8–16 years), Compared with Population Norms

		TGA (*N* = 40)	TGA vs. NORM1
Intelligence (WISC IV)			
Full scale IQ (FSIQ)		97.0 ± 12.9	0.177
Verbal comprehension		101.0 ± 13.6	0.657
Perceptual reasoning		97.5 ± 12.7	0.210
Working memory		98.6 ± 12.2	0.461
Processing speed		92.1 ± 13.4	0.001**
**Academic Attainm. (WIAT)**			
Word reading		101.5 ± 13.0	0.468
Reading comprehension		103.5 ± 13.7	0.114
Spelling		97.0 ± 14.6	0.197
Numerical operations		101.5 ± 17.2	0.570
Mathematical reasoning		99.9 ± 13.7	0.948
**Verbal Fluency (D‐KEFS)**			
Letter fluency		106.8 ± 14.5	0.005**
Category fluency		100.0 ± 14.0	1.000
**Memory (CMS)**			
General memory (MQ)		93.2 ± 18.9	0.025*
Visual immediate memory		100.3 ± 14.6	0.882
Visual delayed memory		93.0 ± 16.6	0.010**
Verbal immediate memory		93.6 ± 17.3	0.024*
Verbal delayed memory		91.7 ± 19.8	0.010**
Mean immediate memory (Vis/Verb)		97.0 ± 12.6	0.134
Mean delayed memory (Vis/Verb)		92.3 ± 14.2	0.001**
Attention		102.2 ± 15.8	0.385
Learning		97.8 ± 14.1	0.332
Delayed recognition		95.4 ± 13.8	0.041*
	**Controls** (*N* = 64)		
**Episodic memory (RBMT)**			
Profile score (/22)	20.1 ± 2.4	18.0 ± 3.3	<0.001**

Data are shown as mean standard scores ± standard deviation (SD).

**, *Statistically significant differences.

*P*‐values are for differences between patients' group mean and the standard mean of 100 (±15) determined by one‐sample t‐test (df = 39; two‐tailed significance).

*P*‐values are for differences between patient and control groups' mean scores: The scores were compared using a Mann‐Whitney U‐test.

Abbreviations: WISC IV, Wechsler Intelligence scale for children, 4^th^ UK Edition; WIAT, Wechsler individual achievement test, 2^nd^ UK edition; D‐KEFS, Delis‐Kaplan executive function system; CMS, Children's memory scale; RBMT, Rivermead behavioural memory test.

Within the processing speed subscales, i.e. coding (mean scaled score, SD, 7.68, 3,15) cancellation (10.25, 3.05), symbol search (9.43, 2.52, data not included in Table 2), coding, a measure of fine motor control and procedural learning was the most affected.

### Correlations Between Hippocampal Volumes and Neuropsychological Function

**Figure 3:**
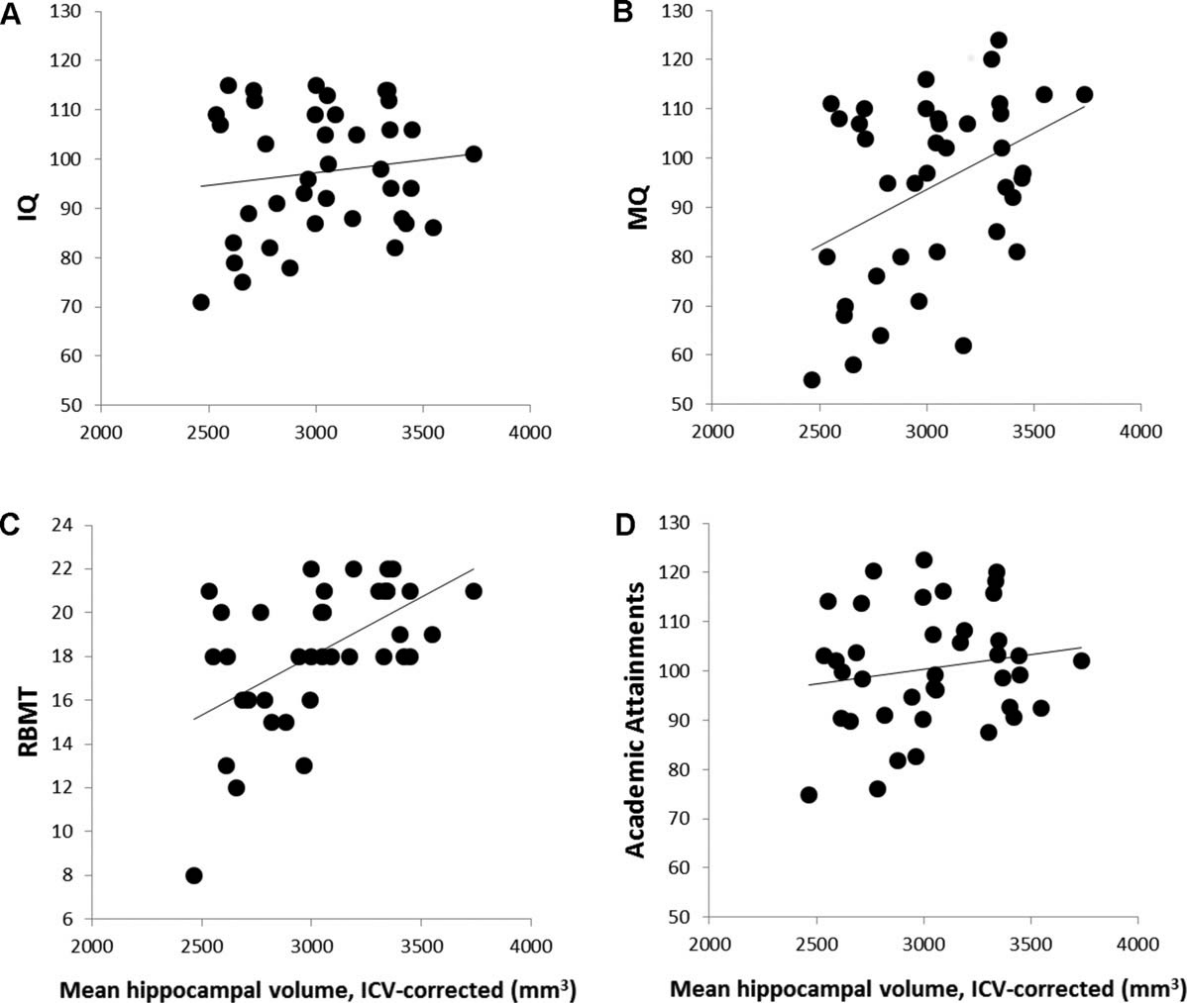
Scatter plots illustrate the correlations between corrected hippocampal volumes (HVs) and neuropsychological function. While HVs correlated with measures of memory function, they were independent of IQ and Academic attainments. **A**. Scatter plot illustrates the poor association between HVs and IQ; **B**. Graph illustrates the high linear correlation of HVs with Memory Quotient; C. Linear correlation between HVs and RMBT, a measure of episodic and spatial memory; **D**. Academic attainments were independent of HVs.

HVs in the TGA group correlated significantly with General Memory (MQ) (Pearson *r* = 0.408, *P* = 0.01, Fig. 3a), immediate verbal memory (Pearson *r* = 0.495, *P <* 0.001), delayed verbal memory (Pearson *r* = 0.397, *P* = 0.012), mean immediate memory (Pearson *r* = 0.399, *P* < 0.012), mean delayed memory (Pearson *r* = 0.388, *P* = 0.015), and RBMT scores (Spearman *ρ* = 0.563, *P* < 0.001, Fig. 3b). In contrast, HV did not correlate with IQ (Fig. 3c), verbal fluency, or academic attainments (Fig. 3d).

### Neuropsychological Function in TGA Patients with Moderate Versus Severe Hippocampal Volume Reduction

Although the TGA group as a whole showed significant HV reduction, this was not true of each group member. Sixteen of the 40 TGA cases (40%) had hippocampal volume reduction greater than 10% (TGAH relative to the NC group (range for TGAH subgroup, −11.64 to −52.64; NC, −18.28 to 15.69). The other 24 TGA cases (TGAN, 60%) had hippocampal volumes comparable to those of the NC group (range −9.72% to 14.65%).

Hippocampal volume reduction of the TGAH group is reflected in their impaired memory ability. Compared to the population mean, patients with TGAH had reduced scores in general memory (MQ) (83, *P* = 0.003), immediate verbal memory (83, *P* < 0.001), delayed visual memory (89, *P* = 0.026), delayed verbal memory (82, *P* = 0.003), and learning (89, *P* = 0.01). In contrast to these memory deficits, compared to the population mean, patients with TGAH had reduced scores in one of the IQ indices (processing speed index = 88, *P* = 0.008), but not significantly reduced scores in the other three IQ indices, or in any of their academic skills, nor in verbal fluency.

Patients with TGAN had no memory deficits. Similarly to TGAH group, however, their performance on the processing speed index was reduced compared to the population mean (95*, P* = 0.028).

When compared to TGAN, TGAH group had reduced scores in General Memory (TGAN mean = 100, TGAH mean = 83, *P* = 0.007), as well as immediate verbal memory (TGAN mean = 101, TGAH mean = 83, *P* = 0.001), delayed verbal memory (TGAN mean = 98, TGAH mean = 82, *P* = 0.014), and learning (TGAN mean = 103, TGAH mean = 89, *P* = 0.003).

### Memory Function in the TGA Patients Relative to IQ

Although the TGA group as a whole showed selective memory deficits, this too was not true of every group member. A criterion for diagnosing memory impairment in individual cases is a significant discrepancy between the IQ‐predicted MQ and the actual MQ (Cohen, 1997). Thirteen of the 40 patients (32%) met this criterion, and so formed a subgroup with a mean discrepancy score of −24 points (actual MQ, 70; predicted MQ, 94), compared with a mean discrepancy score in the remaining patients of +4 points (actual MQ, 104; predicted MQ, 100). The affected subgroup's memory impairment was also reflected in their performance on the Rivermead test compared with the unimpaired group (mean scores of 15.5 vs. 19.1 *P* = 0.002; Mann‐Whitney *U*‐test). Further, parental ratings of difficulty with everyday memory and spatial orientation were higher for the children in the affected subgroup than they were for the other children (100.3 *vs*. 77.1, *P* = 0.060). This deficiency, though not significant, appeared to hinder social interaction according to parental ratings (affected 59.1 *vs*. unaffected 54, *P* = 0.008).

Finally, although each one of this subgroup of patients had significant, and indeed substantial, memory impairment, not each one had significant HV reductions; of the 11 patients with scores below 17 in the Rivermead, eight had abnormally small hippocampi (mean HV reductions of 18%), whereas the mean reductions in the three other cases was only 2.4%.

## DISCUSSION

The cohort of children born with transposition of the great arteries (TGA) and treated with the arterial switch operation (ASO), like the cohort studied earlier that had sustained acute hypoxaemic respiratory failure (AHRF), showed significant memory impairment in association with abnormally small hippocampal volumes. The memory deficiency was observed not only on standardized tests of general memory ability but also on the Rivermead test, which measures memory for everyday events in visual, verbal, and spatial domains, as well as in the parent's ratings of their children's memory problems. Further, the impairment was selective to memory, in that other cognitive abilities (intelligence, academic attainments, and verbal fluency) appeared to be largely unaffected. The findings thus provide additional support for the hypothesis that neonatal hypoxia, hippocampal atrophy, and memory deficit form a causal sequence, with the triggering event in the present cohort being neonatal cyanosis.

Given that cyanosis had occurred in all of the children, a question posed by our findings is why only a subgroup had detectable hippocampal pathology. Whether those affected sustained a greater degree and/or duration of hypoxia than the unaffected, or lacked a form of genetic protection possessed by the others, or were differentially vulnerable for still other reasons is unknown. The possibility of differential susceptibility among the members of the cohort calls out for further research.

An equally important issue is the variable relationship between hippocampal atrophy and memory impairment. Although group comparisons revealed a significant association between these two outcome measures, a case‐by‐case analysis uncovered exceptions. For example, a few patients with HVs comparable to those of the NC group nonetheless had marked memory losses equal to those of patients with substantial hippocampal atrophy, raising the possibility that the former cases had covert extrahippocampal neuropathology or had hippocampal pathology that took some form other than volume reduction. For example, hippocampal gliosis in the CA1 region in response to ischaemia could not have been detected at low magnetic fields or through volume measurements. Also, areas of white matter involving hippocampal connectivity could have been affected. Three cases had HV reduction, but no hint of memory problems (i.e. MQ < IQ discrepancy). MRI‐based measurement of volume is not free of limitations; it is, therefore, difficult to discard whether these are outliers that reflect variability or that compensation could have occurred. However, these are issues that require further research.

It is worth noting that the correlation between hippocampal volumes and immediate verbal memory seems to contradict the extensive literature and clinical evidence supporting the role of the hippocampus in delayed memory, and not so much in immediate memory. However, the tests employed to measure this function in this study are based on free immediate recall of word list and stories, and these are procedures that exceed the demands of working memory capacity. By the time the participant listens to the all the items comprising the list or the story, they have to reconstruct it in mind, and this goes beyond working memory. Even though the results might seem then paradoxical, the results are consistent with previous evidence in that verbal memory that exceeds working memory capacity is vulnerable to hippocampal damage.

Congenital heart diseases are the most common birth defects, occurring in approximately 1% of live births with approximately one million people worldwide currently living with a congenital heart disease. Transposition of the great arteries is one of the most common congenital heart diseases with 20‐30 incidences every 100,000 live births (British Heart Foundation). Our finding that a significant number of cyanotic neonates are at risk of hippocampal injury and subsequent memory impairment, calls for early screening for such damage, and the need to provide diagnosis and support for those affected early in life before the everyday memory problems become entrenched.
